# What the Fighting Man Thinks of War

**Published:** 1917-08-18

**Authors:** Tommy Atkins

**Affiliations:** Hospital


### What the Fighting Man Thinks of War.

A VOICE FROM THE HOSPITAL WARDS.

[We take the following letter from the *Morning Post* because it represents a point of view, which it were well, if the people throughout the country and Empire could clearly grasp and apprehend. Our warriors, when they come home, will put a speedy end to the Pacifists. Meanwhile, every pond in each village should be carefully attended to by the inhabitants, as it will make an excellent bath where Tommy can wash, on Sunday afternoons and at other suitable times, the men who have enjoyed the privilege and protection of living in England—a section of them being of British birth—and have continuously set themselves to further the German cause and malign the British nation. How workmen who have been at the Front or have their children, as we have had ours, fighting for the freedom of the world in this war, can suffer the presence of the few Pacifists of the ignoble type we have in mind as speakers at their meetings, or associate with them in their workshops or in their leisure time, passes our comprehension. If only the working men would take these fellows in hand and treat them as they deserve, it would be better for England and better for all of us, our sons, and the future generation. We hope every subscriber to The Hospital will read and re-read " Tommy Atkins' " letter, and join with us in thanking the Editor of the *Morning Post* for giving it publicity.]

*To the Editor of* The Morning Post.

Sir,—I don't quite know how to begin this letter, because I am not in the habit of writing to newspapers, but I have got something to say, and I guess I can say it just as well without using the classical form.

I am just what is called a " common soldier," who has had nearly three years' steady at the Front, and I—like many of my comrades—am pretty tired of hearing you people at home *talk*; we think it is about time you heard something from *us*, who are doing the bloody work (no, this isn't a swear word, it is just God's truth!), and what *we* think about things.

(*Continued on p.* 405.)

### The Editor's Letter-Box (*continued from p.* 400).

### What the Fighting Man Thinks of War—(*continued*).

First of all, we want to know: are we going to be allowed a voice in the matter of peace terms—we through whose suffering flesh and blood peace will come, if it ever comes at all!—or are you chaps who have sat at home, warm and cosy in the winter, cool and comfy in the summer, going to settle this question to suit yourselves and serve your party interests, without consulting *us* who have fought and bled for you?

No, I'm no Socialist, and still less am I a Pacifist. I am just a man and an Englishman, and I'm out to see fair play. I say what we have fought for through these years, that we *want*, or else there will be trouble somewhere!

You send your Mr. Ramsay Macdonalds and your Mr. Philip Snowdens out into the firing line, and let them see their best pals blown to bits, and all the horrors and the stench that we have been called on to face, day in, day out, through these three—sanguinary, if you like it better so—years; you ask them how *they* like having shrapnel bullets and bits of shells blown into their tender skins (although I think their hides are tougher than their consciences!), and then tell me if they are still clamouring to grant their "dear brethren," the Huns, a lenient peace!!

No! I say, I who have seen all my best pals killed or hideously maimed: I who have been frozen up to the waist in icy water in winter, and baked by a pitiless sun in summer: I who haven't slept in a bed hardly for three years: who have four times been in hospital for what the R.A.M.C. were pleased to call "slight wounds" —because they didn't necessitate the cutting off of a limb! —but I would like your Pacifists to try and see how *they* enjoy the feeling of even "slight wounds"!!—I say it is for *us*, who have fought and endured, to *demand* from the Government who holds office, because *we*, the masses, have put them there! that all our sufferings shall be taken into account, and the blood that has been shed shall be paid for in full. I say if our Government isn't strong enough to keep this small party of Pacifists, those gentle friends of the Germans, in their proper place (and that is, under the heel of all who call themselves Men) then let this Government get out, and we will bring in a new Government that *does* represent the opinions of those who put them in office. We are quite ready to fight this war out to the end, but we are *not* ready to have the hardly-won fruits of victory torn out of our hands by a canting lot of stay-at-home-to-save-their-skins (since they like German phrases!)—Pacifists and Cowards!—Yours, etc.,

TOMMY ATKINS.

—— Hospital, August 7.

[The writer of this letter asks that the name of the hospital from which he writes shall not be disclosed; but we can tell our readers that it is in the London district.—ED. *M.P.*]

